# Results of a multinational survey regarding the diagnosis and treatment of temporomandibular joint involvement in juvenile idiopathic arthritis

**DOI:** 10.1186/1546-0096-12-6

**Published:** 2014-01-25

**Authors:** Ivan Foeldvari, Nikolay Tzaribachev, Randy Q Cron

**Affiliations:** 1 Hamburg Center for Pediatric and Adolescent Rheumatology, Hamburg, Germany; 2 Rheumatology Kid's Clinics, Bad Brahmstedt, Germany; 3 University of Alabama at Birmaingham, Birmingham, AL, USA

## Abstract

**Background:**

Temporomandibular joint (TMJ) involvement occurs in up to 80% of patients with juvenile idiopathic arthritis (JIA). Currently there are no standardized procedures regarding diagnosis and treatment of this common complication of JIA. The aim of the study was to assess the current clinical practices in many countries regarding diagnosis and treatment of TMJ involvement in JIA. Pediatric rheumatologists were asked to fill out a survey with 8 items regarding diagnosis and treatment of TMJ involvement. The survey was distributed over the worldwide pediatric rheumatology electronic list-serve. Data was collected in an Excel spread sheet and analyzed using Excel software.

**Findings:**

Eighty-seven centers responded to the survey between December 2009 and April 2010. All responding centers were actively screening for TMJ involvement. All centers were screening by physical exam, 85 (97%) by history, and 2 (3%) by imaging. Seventy-seven (88%) centers were screening at the first visit and 76 (87%) at each follow-up visit. If imaging was requested, 77% of the centers reported that they asked for MRI, 10% for ultrasound, 9% for CT and 33% for X-ray. The first line treatment of TMJ arthritis was a non-biologic DMARD in 36%, an NSAID in 33%, an intraarticular corticosteroid injection in 26%, and an anti-TNF agent in 5%. Overall, 57 (65%) of the centers were using intraarticular corticosteroid injections as treatment.

**Conclusions:**

TMJ arthritis is common among children with JIA. This survey shows that a wide array of diagnostic and therapeutic approaches is being employed for TMJ disease in 87 international centers. Due to this lack of agreement in how to diagnose and treat this JIA complication, we believe that an expert opinion/consensus statement regarding TMJ arthritis in JIA will likely benefit patients worldwide.

## Background

Temporomandibular joint (TMJ) arthritis occurs in up to 80% of patients with juvenile idiopathic arthritis (JIA) by MRI assessment, but it is asymptomatic in up to 70% of the patients [1,2]. The diagnosis of TMJ involvement in JIA is still difficult [3]. The range of the reported prevalence is wide, and it depends to a large part on the method of assessment of TMJ arthritis. The sensitivity and specificity of the clinical examination by pediatric rheumatologists and different imaging methods leads to a wide range of reported TMJ involvement [4,5]. A recent publication in which even clinically quiet TMJs were imaged by MRI found the highest prevalence of TMJ arthritis [1]. There is even less known about ideal therapy for TMJ arthritis once it is diagnosed in children with JIA.

To date, there is only one small prospective study reporting on the treatment of TMJ arthritis exclusively [6]. In clinical experience TMJs appear to respond less well to the standard of care used to treat other joints. This is reflected in the study populations of Ringold et al. [6] and Arabshahi et al. [7]. In these studies, a large proportion of patients received a DMARD plus anti-TNF treatment, and despite these therapies, patients developed TMJ arthritis. These publications included intraarticular corticosteroid treatment of TMJ arthritis and demonstrated a response in subsets of the JIA patients [6,7], but not all of the treated JIA patients regain a normal mouth opening. These outcomes may reflect timing of the treatments. Currently they are no standardized protocols regarding diagnosis and medical treatment of this common presentation of JIA. To gain more information about the current practice standards regarding diagnosis and medical management of TMJ arthritis in children with JIA, a multinational email survey was conducted.

## Methods

Pediatric rheumatologists, each one representing a unique pediatric rheumatology center, were asked to fill out a survey with 8 items regarding diagnosis and medical treatment of TMJ arthritis. They were asked about the size of the clinic population and the proportion of JIA patients having TMJ arthritis as assessed clinically and by imaging. The questionnaire is attached in the Additional file 1. The survey was distributed over the worldwide pediatric rheumatology electronic list-serve similar to other surveys [8]. Data was collected on an Excel spread sheet and analyzed using Excel software. It was stored on the first author’s computer and simple statistics were applied using Excel.

The data was originally evaluated using only the first 77 centers responding. This data was compared to responses from the eventual total 87 worldwide centers. Reassuringly, neither the answers nor the distribution of the answers changed (data not shown).

## Findings

**Figure 1:**
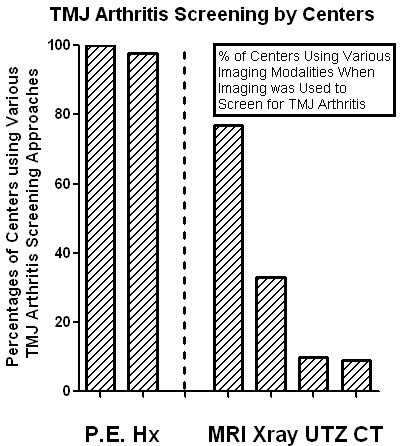
**Screening for TMJ arthritis.** The percentages of centers screening for TMJ arthritis among their JIA cohorts by either physical exam (P.E.) or history (Hx) are shown on the left hand side of the bar graph. Of those centers that used imaging as screening for TMJ arthritis, the percentages of the centers using the various imaging modalities [MRI, Xray (including orthopantomogram), ultrasound (UTZ), CT] are noted on the right hand side of the bar graph. More than one imaging modality per center may be employed.

Eighty-seven centers responded to the survey from December 2009 through March 2010, centers mostly from mainland Europe, United Kingdom, United States, and Canada. This represents about 10% of the participating centers on the pediatric rheumatology electronic list-serve. Forty-three of the centers followed less than 300 patients with JIA, and 44 centers cared for more than 300 patients with JIA. Specifically, 27 centers followed 300 to 499 patients, 12 centers followed 500 to 1,000 patients, and 5 centers followed over 1,000 patients with JIA. All responding centers were actively screening for TMJ involvement (Figure 1).

**Figure 2:**
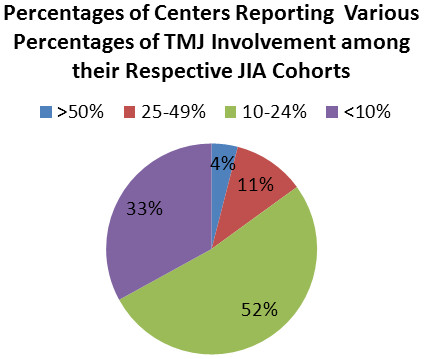
**The perceived prevalence of TMJ arthritis among the JIA cohorts at the various centers.** The percentages of centers reporting >50% of their JIA cohort with TMJ arthritis (blue), 25-49% with TMJ arthritis (red), 10-24% with TMJ arthritis (green), and <10% with TMJ arthritis (purple) are noted in the pie graph.

Eighty-five of the 87 centers screened for TMJ arthritis by history, and all responding centers screened by physical exam. Only 2 centers primarily used imaging to screen for TMJ arthritis. Seventy-seven (88%) centers were screening all patients at the first clinical visit, irrespective of a positive history for TMJ involvement. Seventy-six centers (87%) were screening all patients at each follow-up visit, irrespective of a positive history of TMJ involvement. If imaging for TMJ involvement was requested for a suspicion of TMJ arthritis, 77% of centers asked for MRI, 10% for ultrasound, 9% for computerized tomography (CT), and 33% for standard radiography (X-ray); some centers used more than one method for screening for the arthritis (Figure 1). The centers reported the following prevalence of TMJ arthritis (Figure 2): over 50% with TMJ arthritis in 4% of the centers; between 25 and 49% involvement in 11% of the centers; between 10% and 24% involvement in 52% of the centers; and less than 10% involvement in 33% of the centers.

**Figure 3:**
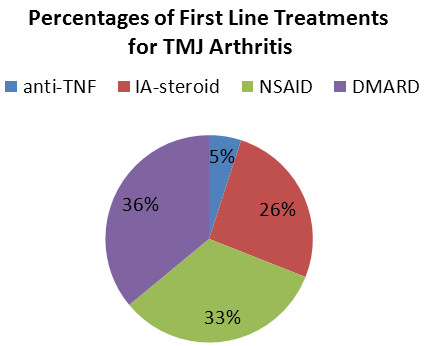
**Distribution of the different first line treatments for TMJ arthritis at the various centers.** The percentages of centers using systemic anti-TNF treatment (blue), intraarticular corticosteroids (red), NSAIDs (green), and non-biologic DMARDs (purple) for first line therapy of TMJ arthritis among their respective JIA cohorts are shown in the pie graph.

The first line medical treatment for TMJ arthritis (Figure 3) was a non-biologic DMARD in 36%, an NSAID in 33%, an intra-articular corticosteroid injection in 26%, and a systemic anti-TNF agent in 5% of the patients. Overall, 57 of the centers (65%) were using intraarticular corticosteroid injections as treatment at some point in their treatment algorithm. Of the 57 centers, 32 (56%) were using imaging as guidance for the intraarticular TMJ injection. Twenty centers provided details about imaging for the TMJ injections. For these 20 centers, MRI was used in 10%, CT in 30%, ultrasound in 45%, and fluoroscopy in 15%. In 7 centers the injection was performed by an oral surgeon or a pediatric dentist; in these centers no information about imaging during the procedure was given.

## Discussion

The aim of this survey was to assess current clinical practice for diagnosis and treatment of TMJ arthritis in children with JIA. TMJ arthritis is common among those with JIA, but a wide array of diagnostic and medical therapeutic approaches are being employed by pediatric rheumatologists according to this survey. It is encouraging that most responders screen for TMJ involvement, but there may be a bias that centers who do not consider TMJ involvement as an important issue did not respond to the survey. It is likely that the centers that frequently consider TMJ involvement in JIA, and how to screen and diagnose TMJ arthritis, may have answered the survey while those centers that are less interested did not.

The prevalence of TMJ arthritis in the different centers appears to be widespread, which likely reflects the different sensitivities of the applied screening methods. Most of the responding centers screened for TMJ arthritis by history and physical examination. We did not ask what items of the history and physical were assessed. A recent study showed that decreased mouth opening and lateral protrusion of the chin seems to be most sensitive clinically [9]. Applied questions for orofacial symptoms have not been previously assessed [10]. Unfortunately, we cannot judge from our data how sensitive the clinical history and the physical examination are for detecting early TMJ involvement.

However, it is remarkable that a large proportion of the centers still use X-ray for imaging of early TMJ arthritis, even though the poor sensitivity of this imaging for early changes of TMJ arthritis is well known. Radiographs appear to be better suited for studying TMJ bony changes later in the disease course [11]. Although ultrasound appears to be not nearly as sensitive as MRI at detecting early TMJ arthritis signs [1,4], ultrasound appears to be still used at some centers for first line imaging [5] for unclear reasons. Thus, a variety of imaging screening methods with different sensitivities for detecting early TMJ arthritis are being used for detection of TMJ arthritis in children with JIA. Unfortunately, in our survey we did not ask if MRI of the TMJ was done with or without gadolinium. A recent study of note suggested that normal children without JIA may have some uptake of gadolinium in the TMJ; however, the significance of this finding is not yet clearly understood [12].

This survey also revealed that first line medical treatment for TMJ arthritis in children with JIA included a wide range of different classes of medications. This might reflect different severities of the treated TMJ arthritis and the different modalities used to detect TMJ arthritis. As the question in the survey clearly asks for the first line treatment, we would assume the response likely reflects treatment of early disease. Also, there was no information about additional joint involvement provided by the responders and treatment described appeared to be directed at TMJ disease.

Intraarticular steroids for TMJ involvement were employed in 65% of the responding centers. It is possible that some centers are hesitant to use intraarticular steroids due to animal model data in rabbits showing arrest of the mandibular growth plate after intraarticular corticosteroid injection [13]. This risk was not confirmed in another animal study or in a study on humans [2,14]. It could also be that some centers do not have the expertise and experience to do these injections. In addition, the imaging modality used for guidance during intraarticular steroid injections into the TMJ was varied. These differences may reflect local availability, ease of various forms of imaging guidance, the requirement for sedation, or even costs involved. Finally, the benefit of intraarticular steroid application is not fully proven yet and is not fully evidence-based [15]. Our survey did not assess physiotherapy and the orthodontic treatment options in the treatment of TMJ arthritis. We also did not assess the use of biologic intraarticular injections for TMJ disease [16]. Future surveys may be valuable in defining these gaps in data and level of detail.

## Conclusions

TMJ arthritis is common among children with JIA. We surveyed 87 pediatric rheumatologists demonstrating a wide array of diagnostic and therapeutic approaches for TMJ involvement in JIA. We are aware that as participation in the survey was voluntary it is likely that physicians more interested in TMJ involvement participated. Due to a lack of agreement in how to diagnose and treat TMJ arthritis in JIA as demonstrated in this survey, we believe that an expert opinion/consensus statement regarding TMJ arthritis in JIA will likely benefit patients worldwide.

## Competing interests

The authors declare that they have no competing interests.

## Authors’ contributions

IF developed the survey with the input of NT and RC. IF sent the survey out, collected, and analyzed the results. IF wrote and revised the manuscript with assistance from NT and RC. All authors read and approved the final manuscript.

## Supplementary Material

Additional file 1 Survey regarding TMJ involvement in Juvenile Idiopathic Arthritis. Click here for file
