# Supplementary material for: Results of a multinational survey regarding the diagnosis and treatment of temporomandibular joint involvement in juvenile idiopathic arthritis
Source: Pediatr Rheumatol Online J. 2014 Jan 25;12:6. doi: 10.1186/1546-0096-12-6 (PMC3910235; doi:10.1186/1546-0096-12-6)
Supplement: Additional file 1 — Survey regarding TMJ involvement in Juvenile Idiopathic Arthritis. [file 1546-0096-12-6-S1.docx]

**Survey regarding TMJ involvement in Juvenile Idiopathic Arthritis**

Dear colleagues, please take 5 minutes of your busy life, to fill out this survey.

1)  Approximately how many patients do you follow with JIA?

        < 300             o         300- 500          o         500 – 1000      o          over 1000        o

2. Which portion of your patients have TMJ involvement:

        <10%            o          10 - 25 %         o        25 – 50% o          over 50%         o

3)  How do you**screen** for TMJ disease?

                  A, by history              Yes      o               No       o

                  B, clinical examination     Yes     o               No       o

                  C, Imaging as screening method, in patients without symptoms         Yes o No       o

                              If  yes, please specify………………………………………

4)  Do you screen at first visit?           Yes      o             No       o

5. Do you screen at each follow up visit?    Yes      o    No       o

6. If you request imaging:

 MRI          o        Ultrasound       o        CT       o

X-ray or orthopantomogram              o

7. What is your first line of treatment

 NSAR    o          DMARD     o      Intraarticulare steroids o

anti-TNF          o

8. if you apply intraarticulare steroids –

       Imaging guidance      yes      o          no        o

If yes, specify……………………………………………………………………….

Please send response to  [sprechstunde@kinderrheumatologie.de](mailto:sprechstunde@kinderrheumatologie.de)   or fax +494020923693

If you have any questions please contact me – Ivan Foeldvari

Thanks for your participation ☺
